# The Immediate Effect of Therapeutic Touch and Deep Touch Pressure on Range of Motion, Interoceptive Accuracy and Heart Rate Variability: A Randomized Controlled Trial With Moderation Analysis

**DOI:** 10.3389/fnint.2018.00041

**Published:** 2018-09-21

**Authors:** Darren J. Edwards, Hayley Young, Annabel Curtis, Ross Johnston

**Affiliations:** ^1^Department of Interprofessional Health Studies, Swansea University, Swansea, United Kingdom; ^2^Department of Psychology, Swansea University, Swansea, United Kingdom

**Keywords:** touch, OMT, interoception, heart rate variability, range of motion

## Abstract

**Background:** There is paucity in the literature regarding the role of the interoceptive pathway through the insular cortex (IC), as well as heart rate variability (HRV) in relation to Osteopathic Manipulative Therapy (OMT) and deep-touch.

**Aims:** The present study investigated whether both OMT treatment and deep-touch (a newly hypothesized treatment option) was effective at altering the interoceptive pathway and HRV, whilst OMT was only expected to be effective for increasing Range of Motion (ROM).

**Methods:** Thirty-five healthy volunteers were randomly allocated into three conditions in a repeated measures crossover design; a control (laying supine on a plinth); deep-touch (head cradling); and an osteopathic mobilization therapeutic technique on the temporomandibular joint (TMJ). Interoceptive accuracy (IAc), HRV, as well as range of motion (ROM) for the TMJ area as well as the cervical spine (Csp) right and left measures were taken pre and post each condition setting.

**Results:** Significant condition effects emerged from the deep-touch and mobilization interventions for IAc where increases were identified through planned comparisons. For the HRV measure (RMSSD), a significant effect emerged in the deep-touch condition (increase) but not in the mobilization or control conditions. ROM did not increase for any condition. IAc correlated with post-ROM outcomes in many cases and HRV moderated some of these relations.

**Conclusion:** These results are discussed in the context of clinical practice, where cranial deep-touch maybe an effective treatment and modulator of the parasympathetic nervous systems, as well as the interoceptive system.

## Introduction

Interoception can be defined as the moment-to-moment representation of all bodily sensations (Craig, 2002), and involves many parts of the body and brain which also includes how the individual reacts and evaluates the sensations (Cameron, 2001). It is considered important in theories of psychology such as emotion (Lange, 1885; Schachter and Singer, 1962; Damasio, 1994; Damasio and Carvalho, 2013) and it can have an effect on cognitive and behavioral functions (Barrett et al., 2004; Critchley et al., 2004; Wiens, 2005; Herbert et al., 2007a), benefiting the attentive process (Matthias et al., 2009), behavioral self-regulation (Herbert et al., 2007b), and decision making (Werner et al., 2009).

Altered interoceptive awareness is associated with a wide range of problematic conditions such as pain and mental health disorders. For example, on the negative side, it is associated with chronic pain (Schmidt et al., 1989) as well as several mental health related problems such as depression and anxiety (Paulus and Stein, 2010), addiction (Naqvi and Bechara, 2010), eating disorders (Pollatos et al., 2008; Herbert and Pollatos, 2014), somatoform disorders (Mirams et al., 2012; Schaefer et al., 2012), and post-traumatic stress disorders (Wald and Taylor, 2008). These negative associations highlight the need to explore this area of interoceptive awareness regulation more closely and this includes the area of manual therapy and pain management.

From a psychophysiological perspective, Craig (2013) defines interoception as the ongoing homeostatic and sensory afferent pathway of the autonomic nervous system (ANS), which carry the signals from small diameter A delta and C primary afferent fibers from all bodily tissue to the brain. The role of slow conduction velocity mechanosensitive c-fibers called c-tactile (CT) afferents in the role of the representation of the body’s physical condition have also been confirmed in a number of neuroscience studies, which have used different touch-based modalities (Essick et al., 1999; Craig, 2002; Craig and Craig, 2009; Löken et al., 2009; Björnsdotter et al., 2010; Fairhurst et al., 2014).

These signals are then decoded and travel through the spine via synaptic relays of lamina I and II, and then to the brain stem homeostatic regions, thalamus, and finally to the anterior insular of the insular cortex (IC) (Craig, 2002, 2003). Findings suggest that there is a CT-based affective homunculus within the IC which becomes active during touch in perinatal osteopathic manipulative therapy (OMT) (McGlone et al., 2017) and is central for multimodal interoceptive integration of bodily awareness information such as touch stimuli (McGlone et al., 2014).

However, despite these interesting findings in relation to the activation of the IC pathway during OMT touch, very little is known about how manual therapy affects the interoceptive system (Bordoni and Marelli, 2017). One type of manually therapy called OMT, is a drug-free medical approach which uses touch, manipulation, and mobilization procedures to diagnose and treat pain. It uses a structured evaluation to diagnose somatic dysfunction of the skull, spine, pelvis, abdomen, upper and lower limbs before applying touched based treatment in the form of massage, manipulation and mobilization (Cerritelli et al., 2015).

The physiological aspects of how this therapy interacts with the body is understood well but is has only been until quite recently that sufficient knowledge has been discovered about the interoceptive system. The exteroceptive system has been studied extensively by manual therapists but the newly understood interoceptive system has been ignored (D’alessandro et al., 2016). This is a shame as perhaps important aspects of etiological mechanisms relating to touch, interoception and pain perception, may be missing from the manual therapists’ toolbox in clinical practice. So, this forms the first aim of this study, to understand whether touch and OMT alter the baseline interoceptive state.

This interoceptive state is referred to as interoceptive accuracy (IAc). It is a measure of interoception and is usually assessed by measuring the individual’s accuracy to perceive their own heart rate (Pollatos et al., 2009). IAc is positively correlated with immediate emotional and pain experience (Pollatos et al., 2012). Higher baseline IAc has been found to be associated with a change in sympathovagal balance where there is a more pronounced parasympathetic decrease and sympathetic excitation (Pollatos et al., 2012). It is also positively correlated to the self-regulation of behavior in situations that are accompanied by somatic and or physiological changes such as physical workload. An example of this is where in one study participants were asked to pedal on a bicycle ergometer for 15 min. Those with higher baseline IAc covered less distance, demonstrated smaller amounts of heart rate change, stroke volume, and cardiac output which was not accounted for levels in their levels of fitness (Herbert et al., 2007b). As IAc seems to regulate behavioral outcomes, it may also regulate ROM outcomes which are also behavioral. So, this forms the second aim of this study, to investigate the degree to which baseline IAc relate to ROM outcomes.

Interestingly, higher heart rate variability (HRV) which is a measure of the parasympathetic nervous system (PNS) is also related to attentional, emotional, and behavioral self-regulation. It is suggested that the vagal tone index measures the efficacy of central-peripheral neural feedback mechanisms (Porges, 1991, 1992; Nyklìček et al., 1997). Specifically, higher baseline vagal tone relates to the ability to self-regulate effectively and to have higher behavioral flexibility in relation to goal directed behavior (Porges, 1992; Thayer and Lane, 2000). This is strange as both IAc and HRV are positively correlated with behavioral self-regulation except that IAc is positively correlated to a sympathetic response and high HRV is a parasympathetic response. So, a third aim of this study is to explore the relationship between IAc and HRV more closely through moderated analysis whereby HRV is explored as a moderator for any IAc relation. In addition to this, as HRV is also involved in behavioral regulation it may relate directly to ROM outcomes as these are behavioral outcomes, so a fourth aim is to explore the relationship between baseline HRV and post intervention ROM.

The impact of OMT on HRV has previously been investigated and has shown that OMT and other manual therapies typically lead to an increase in parasympathetic dominance (Delaney et al., 2002; Henley et al., 2008; Toro-Velasco et al., 2009; Buttagat et al., 2011; Giles et al., 2013). OMT has also been found to effectively counteract the shift of autonomic balance toward a sympathetic prevalence in healthy participants under stressful conditions during a mental challenge task (Fornari et al., 2017), so may have implications for stress reduction and mental health.

What has not been explored in great detail is how many newtons of pressure each OMT technique or touch provides and a detailed record of the vlocity of movement. It is perhaps important to understand the different types of touch and how they may impact on the ANS and IAc systems so to make specific and accurate hypotheses. This is because studies have shown differences in ANS responses based on the type of pressure used, i.e., whether it is light or deep touch. In example of this, in one study, only 0.8 N (newtons of force) and a velocity of 0.2 cm/s was applied to the forearm and thigh areas and led to a significant increase in the sympathetic responding as recorded by electrodermal activity (Olausson et al., 2008). However, Lindgren et al. (2010) have demonstrated that as little as 2.5 newtons (N) of pressure with a velocity of 1–5 cm/sec can lead to a significant increases in PNS activity using a HRV measure. Many other studies have not recorded the exact force and velocity of the touch intervention or OMT technique. For example, in a study by Diego and Field they simply report that massage with moderate force when applied and led to parasympathetic dominance when recorded by a HRV measure (Diego and Field, 2009). Even though the limited specific detail is problematic, the general consensus is that deeper forms of human touch do leads to greater parasympathetic dominance of the ANS (Schleip, 2003).

The touch procedure in this current study involves cradling a person’s head which involves placing pressure on the suboccipital muscles (rear head muscles), as the weight of the head forms its own downward pressure through gravity. As the average head weights approximately 10 lbs (Morningstar, 2002) this converts to 44.48 N of pressure on the suboccipital muscles and hence is a form of deep pressure. So, a fifth aim of the present study is to confirm, as other studies suggest, that this deep touch will lead to a PNS response as indicted by the HRV (RMSSD) measure.

So, in summary, the primary hypothesis is that it is predicted that OMT and deep-touch will affect IAc as they will activate the CT afferent fibers, and thus lead to an increase in IAc. It is predicted that both will increase, but there will be little differences between these conditions as they will active the CT fibers in a similar way. For the second outcome measure, it is hypothesized that the parasympathetic dominated vagal tone (increase in HRV), will be observed after both the deep-touch and OMT conditions, as this has been observed in previous studies (Delaney et al., 2002; Henley et al., 2008; Toro-Velasco et al., 2009; Giles et al., 2013). Again, it is predicted that both will increase, but there will be little differences between them as they will affect the vagal nerve in a similar way. For the third outcome measure, range of motion (ROM), which is a standard OMT measure, it is hypothesized to increase (for all areas) as a result of the OMT but not deep-touch, which is commonly found in OMT research (Whelan et al., 2017). Age, BMI, and gender will be explored as covariates as these could potentially confound the study findings.

In addition to these, baseline HRV and IAc will be correlated with post-ROM outcomes, where it was predicted that both baseline measures will associate with the post-ROM outcomes as they are both involved in behavioral self-regulation. HRV will also be explored as a moderator between pre–post ROM, pre–post IAc and Pre-IAc with post ROM where it is predicted that HRV will moderate these relations.

## Materials and Methods

### Participants and Inclusion/Exclusion Criteria

**FIGURE 1:**
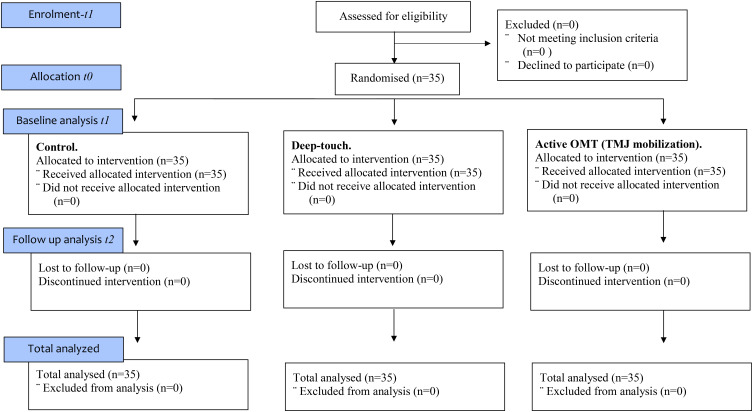
CONSORT flow diagram with three arms and with immediate effects recorded.

A purposive sample of 35 (*M* = 21; *F* = 14) healthy, asymptomatic 1st and 2nd year undergraduate students participated in this study. Participants were included in the trial if they were aged between 18–45 and were English speaking to acquire valid informed consent. Participants with any previous direct trauma to the TMJ, whiplash to the cervical spine (Csp), symptoms of jaw, facial or neck pain, history of Csp disk prolapse, surgical procedures to the Csp, dental surgery or orthodontics within the last year, any form of chronic pain and currently taking any analgesic or anti-inflammatory medication were excluded from the trial. Prior to commencement of the study, participants were randomly assigned to a treatment intervention (control, deep-touch or mobilization) through use of a computer research randomiser (see randomization section) into a sequence of the three condition arms (see CONSORT diagram in **Figure 1**).

### Power Calculation

In order to determine the ideal sample size a G^∗^Power 3.1 (Faul et al., 2007), was conducted where the effect size (assumed moderate) was set to *f* = 0.25; α = 0.05; power = 0.9; number of groups = 1 (within factor) and two covariates (total groups = 3 with two covariates added) by 3 intervention measures. This is appropriate for a 2 × 3 repeated measure, within factors ANCOVA (two covariates), according to Dattalo’s text book on determining sample size using G^∗^Power (Dattalo, 2008). 0.8 power is often suggested in social science studies as adequate, as there should not be more than a 20% probability of making a type 2 error, where the hypothesis is rejected when it should not have been, however, we opted for a higher power of 0.9 with only a 10% chance of making a type 2 error (Cohen, 1988).

### Randomization

Participant crossover randomization was conducted with three condition arms and with six possible sequence combinations; [1, 2, 3]; [1, 3, 2]; [2, 1, 3]; [2, 3, 1]; [3, 1, 2]; [3, 2, 1]. A computer-generated randomiser (Urbaniak and Plous, 2013) was then used to allocate each participant into one of the six condition sequences to mitigate any order effects. Further to this, a 1-week washout period between conditions was employed which again reduced any previous condition order effects.

### Design

This was a repeated measures crossover randomized controlled trial (RCT). The main advantage of the crossover design when compared to a between design is that participants can act as their own controls, and thus variation in participant individual differences between conditions can be removed. In addition to this, a repeated crossover design has greater power when compared with the same sample size but with a between groups design and so can reduce the chances of obtaining a type 2 error (Sibbald and Roberts, 1998).

### Ethical Approval

Ethics were approved through the University Research Ethics Council (REC), which included participant consent, right to withdraw and a full debriefing at the end of the study.

### Blinding

Researcher 1 (R1) conducted the measurements only, whilst Researcher 2 (R2) conduced the intervention only. R1 was blinded to the intervention by leaving the laboratory during the intervention phase. R2 who conducted the interventions was blind to the measurements by leaving the laboratory during the measurement phase.

### OMT Intervention and Location

The area for the OMT mobilization in this present study is called the temporomandibular joint (TMJ) (the jaw joint area), which involves multi-vector guided movement of the TMJ. It is typical to use a measure called ROM when measuring the effectiveness of OMT treatments, which is the ROM of a bodily limb or other appendage at a fixed point (such as the head pointing forward) to a tolerable starching point in a fixed direction (this could be rotating the head left or right, for example) (Whelan et al., 2017).

### Materials and Dependent Variables

#### Venire Calipers and Digital Inclinometer to Measure Range of Motion (ROM)

**FIGURE 2:**
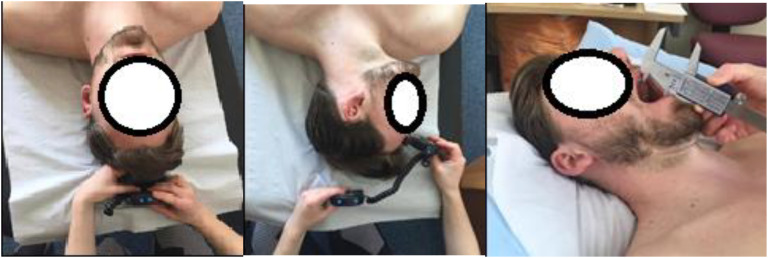
Range of motion (ROM) locations.

Tolerable stretch as an outcome measure has been argued to be most quantifiable and applicable outcome measure during an osteopathic clinical setting to measure the success of an OMT intervention (Butler and Jones, 1991). ROM of the TMJ was assessed using a venire caliper (see **Figure 2**) which has been found to have high intra-assessor reliability (ICC = 0.9–0.98) (Goulet et al., 1998). It was used to establish if the intervention had significant local biomechanical effects on active jaw functionality. An Acumar digital inclinometer was used to assess ROM (see **Figure 2**) at the cervical spine (Csp). Digital inclinometry (DI) has been previously used in several trials assessing cervical, thoracic and lumbar spine ROM where all authors concluded moderate to good inter-rater reliability (ICC = 0.6–0.9) (Prushansky et al., 2010; MacDermid et al., 2014, 2015).

#### Electrocardiogram (ECG) to Measure Interoceptive Accuracy (IAc) Analysis and Heart Rate Variability (HRV)

Interbeat interval data were recorded to measure HRV and interoception using an FDA certified ECG measurement system (BIOPAC MP160) with the patient in a supine position and with conventional Ag/AgCl electrodes. The reference ECG signals were acquired at the sampling rate of 1000 Hz. After being recorded with the BIOPAC’s AcqKnowledge software, R–R interval data were analyzed using Kubios HRV Analysis Software 2.0 (Tarvainen et al., 2002, 2014).

#### Interoceptive Accuracy (IAc) Analysis

In terms of the best possible way to determine IAc, heartbeat detection has emerged as the dominant method (Mandler and Kahn, 1960; Whitehead et al., 1977; Schandry, 1981; Brener and Kluvitse, 1988; Critchley et al., 2004). This is conducted in the form of the heartbeat perception task which is performed according to the Mental Tracking Method (Schandry, 1981) using intervals of 30, 35, 40, and 45 s that are separated by 30 s resting periods. During each trial R–R intervals are recorded, and participants are asked to silently count their heartbeats without the use of an exteroceptive aid (such as taking one’s pulse). At the end of each period participants verbally report the number of counted heartbeats. The participants are not informed about the length of the counting phases nor about the quality of their performance.

#### Heart Rate Variability (HRV)

The recordings were measured at baseline and immediately following each intervention for a period of 5 min. HRV primarily measure PNS, and is an indirect measures of cardiac autonomic balance, where higher values generally indicate higher vagal tone and parasympathetic dominance. One measure of HRV is to use time domain; root mean squared of the successive (R–R intervals) differences (RMSSD) or the R–R interval to measure vagal nerve parasympathetic response, as recommended by the Task Force of the European Society of Cardiology and The North American Society of Pacing and Electrophysiology (Task Force of the European Society of Cardiology and the North American Society of Pacing and Electrophysiology, 1996). Some researchers use spectral analysis of the time series by converting it into a frequency domain such as high frequency (HF) measures for parasympathetic vagal activity and low frequency (LF) for sympathetic activity (Pagani et al., 1984, 1986), however, other researchers suggest that the LF/HF ratio does not accurately measure cardiac sympathetic-vagal balance (Billman, 2011, 2013). In the present study, the time domain root mean square of the standard differences (RMSSD) is the preferred measure for vagal tone but the LF/HF ratio was also explored.

### Procedure and Experimental Conditions

Following inspection of the inclusion and exclusion criteria, and a completed consent form, participants were randomized into one of the six condition sequences (see randomization). The following conditions were then conducted with a 1-week washout period between them, based on those determined randomized sequences, as follows:

#### OMT Mobilization

**FIGURE 3:**
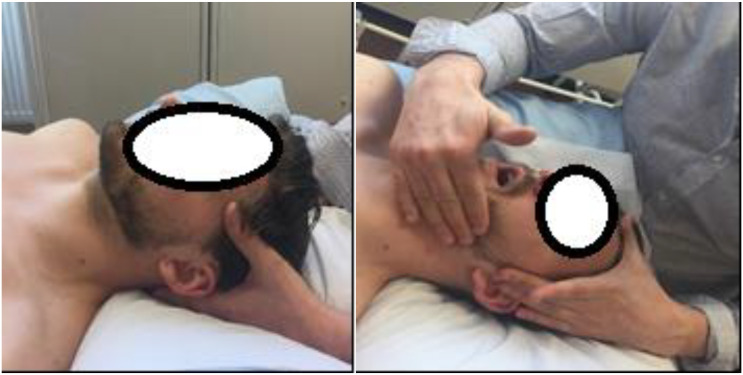
Cranial deep-touch on the left and OMT technique on the right.

All baseline outcome readings (Interoception, HRV, and ROM) and demographic data (in the case that this was the first condition they participated in) were initially assessed by R1. Following this, R1 left the room and R2 entered to conduct the mobilization intervention. Participants in this condition were told the following; ‘Today you will be receiving an osteopathic technique directed toward your jaw joint. Breath normally and relax^1^.’ In a supine position with a pillow supporting the head and neck, R2 then instructed each participant to open and relax their mouth with broad contact over the body and ramus of the participants’ mandible. R2 followed by mobilizing the TMJ into combined vectors of elevation, depression, protraction, retraction and medial and lateral gapping (see **Figure 3**). The mobilization was carried out for a period of 90s. Following this, R2 left the room and R1 returned to measure post-intervention outcome measures (Interoception, HRV, and ROM).

#### Deep-Touch Treatment

All baseline outcome readings (Interoception, HRV, PPT and ROM) and demographic data (in the case that this was the first condition they participated in) were initially assessed by R1. Following this, R1 left the room and R2 entered to conduct the deep-touch intervention. Participants in the deep-touch treatment group were told the following; ‘Today you will be receiving an osteopathic technique. Breath normally and relax.’ R2 instructed the patient to lie supine, with a pillow supporting the head. The deep-touch technique consisted of R2 gently placing both hands over the base of the head, lightly palpating the sub-occipital musculature for a period of 90s (see **Figure 3**). Following this, R2 left the room and R1 re-entered to reassess the outcome measures (Interoception, HRV, and ROM).

#### Control Condition

All baseline outcome readings (Interoception, HRV, PPT, and ROM) and demographic data (in the case that this was the first condition they participated in) were initially assessed by R1. Participants in the control group were told the following, ‘Please lay on the plinth until instructed otherwise, breath normally and relax.’ The participants lay supine on the plinth with their head on a pillow for 90s and then were instructed to sit up. R1 then re-assessed the outcome measures (Interoception, HRV, and ROM).

### Statistical Analysis

All data analysis was conducted through IBM’s SPSS software version 23. Firstly, to correct for positive skewed distributions (when identified through a significant Shapiro–Wilk test), a logarithmic transform (log 10) was used before proceeding with the main analysis to ensure a normal distribution. Correcting non-normality can be made through transforming the data, where it is suggested (Field, 2009) that correcting distributional problems through transformation does not change the statistical relationship between variables. In order to choose the type of transformation, there are some considerations, such as direction of skewness and whether the data contains a value of zero (Field, 2009). Once complete, normality tests were again conducted through Shapiro–Wilk tests where a normal distribution is indicated by a non-significant finding (*p* > 0.05) and thus justifies the use of parametric tests.

For the pre-processing of the HRV data, this was first visually inspected for artifacts caused by ectopic beats, and poor conductivity. A very low correction threshold was chosen for artifact correction (0.45 from local average) so not to distort natural variability. Less than 1% of beats were identified as artifacts and no cases were removed from the analysis. RMSSD was than calculated through the Kubios software which utilized the MATLAB R2011 software.

For the pre-processing of the IAc data, the following transformation was used on the perceived and actual heart beats as used in other studies (Mallorquí-Bagué et al., 2014) whereby a higher score indicates a higher IAc: $1-\frac{\left|n\;beats_{actual}-n\;beats_{reported}\right|}{(n\;beats_{actual}+n\;beats_{reported})/2}$. This was conducted through Microsoft Excel (Microsoft Office 365).

Quantitative data was then analyzed through IBM’s SPSS software version 23, using six separate, two (baseline/after intervention) by three (OMT/deep-touch/control) general linear models, consisting of a repeated measures analysis of covariance (ANCOVA), for each of the three separate areas (TMJ, Csp – left, and Csp – right) for ROM and for the two measures of IAc and HRV (time domain index – RMSSD, as well as the frequency domain index – LF/HF ratio). BMI, age and gender were covariates in the model. When interactions were significant appropriate *post hoc* tests were conducted to determine the nature of the interaction. In addition to this, a series of bivariate correlations were conducted exploring the relation between baseline IAc and RMSSD with post-ROM outcomes for each of the three areas (a total of six). Finally, moderation analysis was conducted using the SPSS PROCESS macro by Andrew Hayes (Hayes, 2013) to explore whether RMSSD moderated the relations between baseline ROM with post-ROM; baseline IAc with post-IAc and baseline IAc with post-ROM outcomes.

## Results

The demographics (mean and standard deviation) of the population were recorded as; Age (Years) = 21.52 (5.12); Weight (KG) = 73.47 (18.01); Height (M) = 1.72 (0.16); and BMI = 24 (5.42). See **Table 1** for the descriptive statistics of the mean and standard deviations of the ROM measure, and **Table 2** for the mean and standard deviations of the HRV and IAc measures.

**Table 1:** Mean, standard deviation (SD) of the scores for ROM at pre and post condition points in time.

Area and condition	Pre-intervention Mean (*SD*)	Post-intervention Mean (*SD*) ROM
TMJ (control)	37.95 (6.21)	38.08 (7.25)
TMJ (deep-touch)	41.85 (9.34)	40.62 (9.90)
TMJ (OMT mobilization)	41.57 (8.54)	41.02 (6.32)
Csp-right (control)	83.83 (9.27)	85.33 (8.97)^∗^
Csp-right (deep-touch)	79.62 (7.89)	81.05 (8.05)
Csp-right (mobilization)	81.47 (7.28)	81.57 (7.28)
Csp-left (control)	81.43 (7.37)	80.60 (8.01)
Csp-left (deep-touch)	78.83 (6.44)	79.02 (7.51)
Csp-left (OMT mobilization)	75.30 (9.75)	76.62 (9.09)


*Note that ^∗^ indicates the conditions where there was a significant difference between pre and post scores (p < 0.05).*

**Table 2:** Mean, standard deviation (SD) of the scores for RMSSD and IAc outcome measures at pre and post condition points of time.

Condition	Pre-intervention Mean (*SD*) HRV	Post-intervention Mean (*SD*) HRV	Pre-intervention Mean (*SD*) IAc	Post-intervention Mean (*SD*) IAc
Control	65.68 (90.51)	71.49 (86.97)	0.78 (0.17)	0.79 (0.19)
Deep-touch	50.60 (39.63)	67.94 (51.65)^∗∗^	0.71 (0.32)	0.83 (0.28)^∗^
OMT mobilization	44.22 (20.98)	41.74 (17.98)	0.73 (0.33)	0.84 (0.24)^∗∗^


*Note that ^∗^ indicates the conditions where there was a significant difference between pre and post scores (p < 0.05) and ^∗∗^ indicates that was significant to p < 0.01.*

### Inferential Statistics

#### Interoceptive Accuracy (IAc)

**FIGURE 4:**
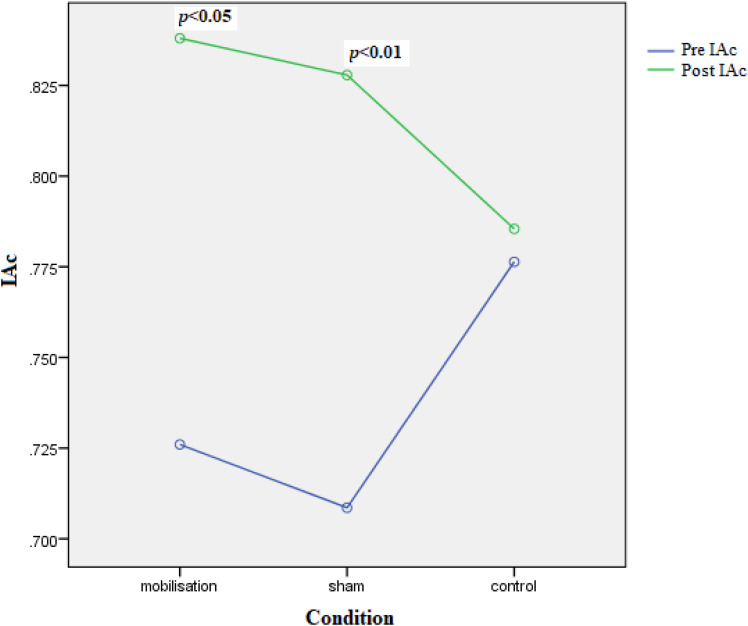
Interaction between pre–post (time) IAc and condition. Significance shows pre–post paired comparisons for each condition where significant.

IAc did not differ according to the covariates BMI [*F*(1,29) = 0.187, *p* = 0.669, $\eta_{p}^{2}$ = 0.006], gender [*F*(1,29) = 0.197, *p* = 0.660, $\eta_{p}^{2}$ = 0.007], or age [*F*(1,29) = 1.654, *p* = 0.209, $\eta_{p}^{2}$ = 0.010]. Although the interaction Time by Intervention did not reach significance [*F*(2,29) = 2.287, *p* = 0.120, $\eta_{p}^{2}$ = 0.136] (see **Figure 4**), *post hoc* Bonferroni comparisons (pre–post interventions) identified that IAc significantly increased in those receiving the mobilization [mean difference = 0.108 (standard error = 0.05), *p* < 0.04] and deep-touch [mean difference = 0.120 (standard error = 0.04), *p* < 0.01] interventions. This increase did not occur in the control condition [mean difference = 0.009 (standard error = 0.05), *p* = 0.857].

#### HRV (RMSSD)

**FIGURE 5:**
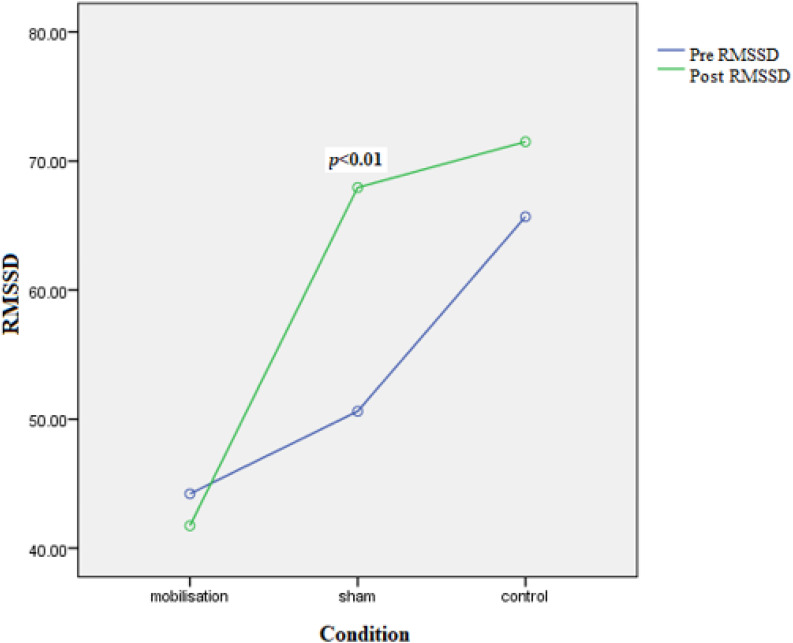
Interaction between pre–post (time) RMSSD and condition. Significance shows pre–post paired comparisons for each condition where significant.

None of the covariates were significant: BMI [*F*(1,29) = 0.559, *p* = 0.462, $\eta_{p}^{2}$ = 0.019], Gender [*F*(1,29) = 0.233, *p* = 0.640, $\eta_{p}^{2}$ = 0.008], and age [*F*(1,29) = 0.416, *p* = 0.524, $\eta_{p}^{2}$ = 0.014]. However, the interaction Time by Intervention was significant [*F*(2,29) = 3.575, *p* < 0.041, $\eta_{p}^{2}$ = 0.198] (see **Figure 5**); again, a significant increase (pre–post interventions) was observed in the deep-touch condition [17.64(4.63), *p* < 0.01] but not in the mobilization [2.48(5.44), *p* = 0.651] or control conditions [5.42(4.89), *p* = 0.277]. To check the reliability of the time domain index RMSSD, then pre and post measures of this for all conditions were correlated with the frequency domain index HF (another index of parasympathetic dominance) and were found to be highly correlated (all were, *p* < 0.05).

#### HRV (LF-HF Ratio)

There were no significant covariates: BMI [*F*(1,29) = 0.100, *p* = 0.754, $\eta_{p}^{2}$ = 0.003], Gender [*F*(1,29) = 0.228, *p* = 0.637, $\eta_{p}^{2}$ = 0.008], and age [*F*(1,29) = 0.027, *p* = 0.872, $\eta_{p}^{2}$ = 0.001]. The interaction Time by Intervention was also not significant [*F*(2,29) = 0.339, *p* = 0.715, $\eta_{p}^{2}$ = 0.023] and neither were the *post hoc* comparisons (all *p* > 0.05).

#### ROM – TMJ

There were no significant effects: Time by Intervention [*F*(2,29) = 0.729, *p* < 0.491, $\eta_{p}^{2}$ = 0.048], BMI [*F*(1,29) = 0.389, *p* < 0.538, $\eta_{p}^{2}$ = 0.013], gender [*F*(1,29) = 0.2.223, *p* < 0.147, $\eta_{p}^{2}$ = 0.071], age [*F*(1,29) = 0.243, *p* < 0.626, $\eta_{p}^{2}$ = 0.008].

#### ROM – CSP Right

Older participants had reduced ROM [*F*(1,29) = 5.131, *p* < 0.031, $\eta_{p}^{2}$ = 0.150] but no associations were observed with BMI [*F*(1,29) = 0.405, *p* < 0.530, $\eta_{p}^{2}$ = 0.014] or gender [*F*(1,29) = 0.099, *p* < 0.755, $\eta_{p}^{2}$ = 0.003]. Those in the control condition had a significant increase in ROM [2.94(1.34), *p* < 0.036]; an effect not observed in the deep-touch [2.40(1.26), *p* = 0.068] or mobilization [1.05(1.49), *p* = 0.486] conditions. The interaction Time by Intervention was not significant [*F*(2,29) = 0.457, *p* < 0.638, $\eta_{p}^{2}$ = 0.031].

#### ROM – CSP Left

The interaction Time by Intervention was not significant [*F*(2,29) = 1.237, *p* < 0.305, $\eta_{p}^{2}$ = 0.079] as were all simple effects. Older participants had reduced ROM [*F*(1,29) = 7.175, *p* < 0.012, $\eta_{p}^{2}$ = 0.189] but BMI [*F*(1,29) = 0.002, *p* < 0.969, $\eta_{p}^{2}$ = 0.000] and gender [*F*(1,29) = 1.379, *p* < 0.250, $\eta_{p}^{2}$ = 0.045] had no influence.

#### Relation Between Baseline HRV (RMSSD) With Post Condition ROM and IAc

There were very few significant relations between baseline RMSSD and post-ROM outcomes. Baseline RMSSD mobilization with post-ROM TMJ mobilization was just outside of significance and in a positive direction (*r* = 0.259, *p* = 0.084); Baseline RMSSD sham with post-ROM TMJ sham was not significant but in a positive direction (*r* = 0.221, *p* = 0.129); Baseline RMSSD control with post-ROM TMJ control was significant but in a negative direction (*r* = -0.306, *p* < 0.05). Baseline RMSSD mobilization with post-ROM Csp right mobilization was not significant but in a positive direction (*r* = 0.152, *p* = 0.212); and so was Baseline RMSSD sham with post-ROM Csp right sham (*r* = 0.129, *p* = 0.257); Baseline RMSSD control with post-ROM Csp right control was not significant but was in a negative direction (*r* = -0.017, *p* = 0.462). Baseline RMSSD mobilization with post-ROM Csp left mobilization was not significant but in a positive direction (*r* = 0.256, *p* = 0.086); this was the same for Baseline RMSSD sham with post-ROM Csp left sham (*r* = 0.002, *p* = 0.496); Baseline RMSSD control with post-ROM Csp left control was significant and in a negative direction (*r* = -0.357, *p* < 0.05); Baseline RMSSD mobilization with post-IAc mobilization was not significant (*r* = -0.190, *p* = 0.157); however, baseline RMSSD sham with post-IAc sham was significant and in a positive direction (*r* = 0.453, *p* < 0.05); Baseline RMSSD control with post-IAc control was not significant but was in a negative direction (*r* = -0.018, *p* = 0.461).

#### Relation Between Baseline IAc and Post Condition ROM

A series of bivariate correlations were also used to also explore the relation between baseline IAc and post-condition ROM, which showed these were several significant relations. Baseline IAc mobilization with post-ROM TMJ mobilization was significant and negatively related (*r* = -0.423, *p* < 0.01).

Baseline IAc sham post-ROM TMJ sham was not significant (*r* = 0.018, *p* = 0.464). Baseline IAc control with post-ROM TMJ control was significant and in a negative direction (*r* = -0.358, *p* < 0.05). Baseline IAc mobilization with post-Csp right mobilization was significant and in a negative direction (*r* = -0.529, *p* < 0.001). Baseline IAc sham with post-Csp right sham was also significant and in negative direction (*r* = -0.410, *p* < 0.01). Baseline IAc control with post-Csp right control was not significant (*r* = -0.153, *p* = 0.198). Baseline IAc mobilization with post-Csp left mobilization was significant and in a negative direction (*r* = -0.509, *p* < 0.01); and so was baseline IAc sham with post-Csp left sham (*r* = -0.364, *p* < 0.05); as well as baseline IAc control with post-Csp left control (*r* = -0.423, *p* < 0.01).

#### HRV (RMSSD) as a Moderator of Change in Pre–post Condition ROM and IAc

As can be seen in **Table 3**, RMSSD significantly moderated the relation between pre–post IAc for the mobilization and sham conditions but not for control. RMSSD did not moderate any of the pre–post ROM conditions and for any of the areas.

**Table 3:** Regression associations between pre and post ROM outcomes as well as pre and post IAc outcomes, where HRV (RMSSD) is used as a moderator (beta coefficient).

Comparison (IV and DV)	*F*-value (*df*)	*p*-value of regression model	*R*^2^	*B* (moderator)	*p*-value of moderator
Pre–post ROM TMJ mob	46.701 (3, 31)	<0.001	= 0.844	= -0.005	= 0.217
Pre–post ROM TMJ sham	39.746 (3, 31)	<0.001	= 0.890	= -0.0002	= 0.951
Pre–post ROM TMJ con	37.395 (3, 31)	<0.001	= 0.736	= -0.0059	= 0.5807
Pre–post ROM CspR mob	41.438 (3, 31)	<0.001	= 0.827	= -0.0002	= 0.9695
Pre–post ROM CspR sham	26.375 (3, 31)	<0.001	= 0.767	= 0.072	= 0.141
Pre–post ROM CspR con	24.779 (3, 31)	<0.001	= 0.719	= -0.001	= 0.9777
Pre–post ROM CspL mob	22.457 (3, 31)	<0.001	= 0.722	= -0.001	= 0.825
Pre–post ROM CspL sham	15.389 (3, 31)	<0.001	= 0.658	= 0.009	= 0.891
Pre–post ROM CspL con	19.591 (3, 31)	<0.001	= 0.669	= -0.003	= 0.583
Pre–post IAc mob	113.363 (3, 31)	<0.001	= 0.929	= 0.126	<0.0001
Pre–post IAc sham	53.752 (3, 31)	<0.001	= 0.870	= -0.0135	<0.01
Pre–post IAc control	0.916 (3, 31)	= 0.446	= 0.086	= 0.0004	= 0.962


*pre–post, association between pre and post measure; mob, mobilization; sham, sham; con, control; B, unstandardized beta coefficient of the moderator; IV, Pre-measure; DV, post-measure.*

#### HRV (RMSSD) as a Moderator of Change in Pre IAc and Post Condition ROM

As can be seen in **Table 4**, RMSSD moderated the relation between pre-IAc and post-ROM for TMJ mob, TMJ control, Csp right control, Csp left sham, Csp control sham.

**Table 4:** Regression associations between pre-IAc and post-ROM outcomes, where HRV (RMSSD) is used as a moderator (beta coefficient).

Comparison (IV and DV)	*F*-value (*df*)	*p*-value of regression model	*R^2^*	*B* (moderator)	*p*-value of moderator
Pre-IAc-post ROM TMJ mob	5.798 (3, 31)	<0.01	= 0.401	= 0.573	<0.01
Pre-IAc-post ROM TMJ sham	1.065 (3, 31)	= 0.383	= 0.118	= 0.589	= 0.199
Pre-IAc-post ROM TMJ con	5.59 (3, 31)	<0.01	= 0.366	= 0.719	<0.05
Pre-IAc-post ROM CspR mob	3.579 (3, 31)	<0.05	= 0.292	= -0.162	= 0.509
Pre-IAc-post ROM CspR sham	2.159 (3, 31)	= 0.119	= 0.213	= 0.371	= 0.259
Pre-IAc-post ROM CspR con	7.788 (3, 31)	<0.001	= 0.446	= 1.359	<0.001
Pre-IAc-post ROM CspL mob	3.471 (3, 31)	<0.05	= 0.286	= -0.197	= 0.504
Pre-IAc-post ROM CspL sham	3.777 (3, 31)	= 3.777	= 0.321	= 0.694	<0.05
Pre-IAc-post ROM CspL con	12.885 (3, 31)	<0.001	= 0.571	= 0.839	<0.001


*pre–post, association between pre and post measure; mob, mobilization; sham, sham; con, control; B, unstandardized beta coefficient of the moderator; IV, Pre-measure; DV, post-measure.*

## Discussion

This study sought out to investigate whether both the OMT treatment and deep-touch (a newly hypothesized treatment option) would be effective at altering the IAc, HRV with little difference between them, whilst OMT would be effective for the increased ROM (as this was a specific measure of OMT effectiveness).

We did not find what we were expecting in some cases. Hypothesis one was found to be true, where a significant increase in IAc was observed for the deep-touch and mobilization conditions (but this was only identified through the pre–post Bonferroni tests). Hypothesis two was not found to be true, and the null was accepted as there was a significant increase in RMSSD for the deep-touch condition only and not the OMT treatment as predicted. Hypothesis three was also found to be false, and the null was accepted as there was no increase in ROM for the OMT condition (or deep-touch).

In addition to these, there were very few associations which were significant between RMSSD and post-ROM outcomes. One of these was a negative correlation between baseline RMSSD control with post-ROM TMJ control; another was a negative correlation between baseline RMSSD control with post-ROM Csp left control. For the relations between RMSSD and IAc, there was only a positive correlation between baseline RMSSD sham with post IAc sham. Many of the IAc scores correlated with post-condition ROM scores. These included negative correlations for the following; baseline IAc scores with post-ROM mobilization TMJ; with post-ROM TMJ control; post-ROM Csp right mobilization; post-ROM Csp-right sham; post-ROM Csp left mobilization; post-ROM Csp left sham; post-ROM Csp left control.

As baseline IAc demonstrated many significant associations with ROM outcomes, RMSSD was also explored as a moderator between the associations of pre–post ROM, pre–post IAc, and pre-IAc with post ROM. RMSSD did not moderate pre–post ROM for any of the conditions or areas, but it did moderate pre–post IAc mobilization and sham conditions, as well as pre-IAc and post-ROM TMJ mobilization and control conditions, pre-IAc with post-ROM CspR control, pre-IAc and post-ROM CspL sham and control conditions.

The significant increase of HRV in the deep-touch condition only and not in the OMT condition was unlike other studies which found a significant increase after the OMT intervention. The reason why the OMT condition did not lead to an increase in RMSSD is perhaps because this was a mobilizing of the jaw joint intervention (rotating the jaw joint) rather than a stroking of the skin intervention such as soft tissue massage as conducted in, for example, the Toro-Velasco study (Toro-Velasco et al., 2009). As for the significant increase in the deep-touch condition, this was not surprising as other studies have found that deep touch pressure has led to a PNS response (Lindgren et al., 2010; Chen et al., 2013).

As light and deep touch seem to affect the ANS system differently, where light touch of only 0.8 N of pressure and a velocity of 0.2 cm/s on the forearm and thigh leads to a sympathetic response (Olausson et al., 2008). Whilst, as little as 2.5 N of pressure with a velocity of 1–5 cm/s (Lindgren et al., 2010) can lead to a significant increase in PNS. Then further investigating the specific amounts of pressure and velocity seem to be very important and more studies should explore this in more detail. Unfortunately, many studies have not recorded this and simply refer to ‘light,’ ‘moderate,’ and ‘deep’ pressure with no specific criteria, such as in a study by Diego and Field (2009). This can be confusing and may lead to inaccurate hypothesis testing between laboratories as one group of researchers may be referring to an intervention as ‘light’ pressure whilst another group of researcher may refer to the same intervention as ‘moderate’ pressure. Therefore, an accurate index of these is needed.

The correlations also produced some interesting findings which showed that in nearly all cases IAc was significantly negatively related with post-ROM outcomes, which means that potentially IAc is a reliable predictor for post-intervention ROM outcomes. Perhaps equally interesting is that RMSSD moderated some of these relations, which shows that HRV scores may strengthen the IAc post-ROM relation outcomes and therefore increase the ability to predict ROM outcomes.

There were also some inconsistencies in the literature (mentioned in the introduction) regarding behavioral self-regulation where both increases in IAc and HRV are related to increased behavioral self-regulation. The inconsistency is that high IAc involves a sympathetic response whilst higher HRV is a parasympathetic response, which of course counterintuitive. The findings of this present study does not claim to explain this inconsistency but it has identified HRV as a possible moderator of the relation between IAc and post-ROM outcomes as well as pre–post IAc mobilization and sham outcomes. Further studies would be needed to specifically explore these relations in the context of behavioral self-regulation, perhaps where HRV is explored as a moderator to perhaps explain these inconsistencies.

The increase in IAc after the deep-touch and OMT demonstrates that touch has an effect not just with deep-touch but also with this particular OMT technique and in this location. Therefore, maybe osteopathic practitioners should be more aware of this general effect in practice, and more research should focus in this area. Awareness is increasing as osteopathic practitioners are beginning to realize that they are missing knowledge in relation to the emotional body through the interoceptive system (Bordoni and Marelli, 2017). However, these present findings support some growing evidence that touch may play an interoceptive role, perhaps in the form of C-fibers (CT afferents) which can also trigger a sense of wellbeing (Schleip and Jäger, 2012).

Accordingly, these findings could translate into a clinical setting of pain management delivered in the form of OMT and other manual therapy. For instance, in a recent systematic review (Di Lernia et al., 2016) it was suggested that lower IAc was more commonly associated with chronic pain (Di Lernia et al., 2016). So, if we understand how to modulate IAc through gentle-touch or OMT in a way which leads to increases, then perhaps this will lead to a decrease in overall chronic pain. In addition to this, if we can also change HRV in a way which creates a stronger moderating effect of IAc, then this may lead to improved clinical outcomes too. More research in this area is of course needed to explore the relation between IAc and HRV (perhaps as a moderator) over a number of interventions, different levels of pressure and with different velocities of applied intervention movement.

There are also important questions around chronic pain and the co-morbidity of psychological health problems. Recently, a study was conducted which showed that OMT reduced chronic pain related psychological issues such as anxiety and mental health dysfunction, but was less effective for fear avoidance and depression (Edwards and Toutt, 2018). Understanding how to best modulate interoception may have a positive impact on some of the pain-associated mental health conditions as well as the physical pain. For instance, we know that lower interoceptive accuracy have been associated with greater illusion of embodiment and alexithymia which is a struggle to identify one’s emotions (Murphy et al., 2017). Psychological disorders such as anxiety have been associated with higher IAc (Mumford et al., 1991). So, as interoception is deeply connected with psychological processing and is used as an information channel representing one’s inner bodily sensation and internal somatic processes (Tsakiris et al., 2011), then better regulation of this channel could lead to better psychological outcomes. Likewise, understanding how HRV moderates these relations could also be very important in improving mental health outcomes.

The vagal nerve (not just as a moderator for IAc) seems to be closely linked to psychological function and positive mental health outcomes. For example, several trials have demonstrated that individuals with anxiety, obsessive compulsive disorders (OCD) and components of chronic pain exhibit lower baseline HRV (Pittig et al., 2013; Chalmers et al., 2014; Koenig et al., 2016; Tracy et al., 2016), which demonstrates that people with emotional disorders or chronic pain have increased sympathetic activity. In addition to this, many studies have demonstrated that manual therapy in general leads to increased HRV, thus an increase in parasympathetic activity (Toro-Velasco et al., 2009; Yang et al., 2013) which may therefore have a positive mental health affect. The relationship between vagal tone and interoception is not understood, but both of these psychophysiological pathways seem to be deeply connected to positive or negative mental health outcomes.

On the whole, this study offers some interesting findings in relation to OMT, touch and its impact on ROM, IAc and HRV. From the literature, it is clear that IAc and vagal tone have a very complex relation with touch, pain perception as well as affective mental health states. However, further integration studies are needed. Integrating biomechanical, spinal, peripheral and neurophysiological components into a single model maybe needed for a complete understanding of how touch and OMT impact psychophysiological outcomes of pain perception, vagal tone and interoception. This has also been supported by previous researchers (Bialosky et al., 2009). Before this is achieved though, further research needs to focus more on the effect of OMT and touch on brain function more generally, as there has been very few of these studies.

In perhaps the only OMT-neuroscience study of this kind (Fryer and Pearce, 2012), a high velocity and low amplitude thrust technique on the lumbosacral joint was found to decrease corticospinal and spinal reflex excitability measured by TMS and EMG, suggesting that at the spinal level, there was an inhibitory effect. But this tells us little about the impact of OMT on IAc and HRV components, or its relation to the IC. In addition to this, the relation between IAc and HRV needs to be explored more closely, perhaps focusing on HRV as a moderator for IAc and post-ROM outcomes and integrating more general studies of behavioral self-regulation. This work represents some exciting results about the relation between deep-touch, OMT, IAc and HRV and it is hoped that more focus will be allocated to these in the future.

## Compliance With Ethical Standards

Ethical approval: All procedures performed in studies involving human participants were in accordance with the ethical standards of the institutional and/or national research committee and with the 1964 Helsinki Declaration and its later amendments or comparable ethical standards. Informed consent was obtained from all individual participants included in the study.

## Author Contributions

DE, HY, RJ, and AC designed the study. DE wrote the majority of the paper with contributions from HY and AC. HY and DE conducted the majority of the statistical analysis. AC contributed to the research design and some aspects of the writing of this manuscript including some aspects of the statistical analysis.

## Conflict of Interest Statement

The authors declare that the research was conducted in the absence of any commercial or financial relationships that could be construed as a potential conflict of interest.

## Footnotes

1 Note, that it was important that the participants were told that this was an osteopathic technique so as to remove any potential expectation bias when comparing the results with the OMT condition.
