# Corrigendum: The Immediate Effect of Therapeutic Touch and Deep Touch Pressure on Range of Motion, Interoceptive Accuracy and Heart Rate Variability: A Randomized Controlled Trial With Moderation Analysis

**DOI:** 10.3389/fnint.2020.00028

**Published:** 2020-05-20

**Authors:** Darren J. Edwards, Hayley Young, Annabel Curtis, Ross Johnston

**Affiliations:** ^1^Department of Interprofessional Health Studies, Swansea University, Swansea, United Kingdom; ^2^Department of Psychology, Swansea University, Swansea, United Kingdom

**Keywords:** touch, OMT, interoception, heart rate variability, range of motion

Annabel Curtis was not included as an author in the published article. The author list has been corrected above and the updated Author Contributions Statement appears below.

## Author Contributions

DE, HY, RJ, and AC designed the study. DE wrote the majority of the paper with contributions from HY and AC. HY and DE conducted the majority of the statistical analysis. AC contributed to the research design and some aspects of the writing of this manuscript including some aspects of the statistical analysis.

The authors apologize for this error and state that this does not change the scientific conclusions of the article in any way. The original article has been updated.

